# Doxapram Hydrochloride Aggravates Adrenaline-Induced Arrhythmias Accompanied by Bidirectional Ventricular Tachycardia

**DOI:** 10.1155/2014/212045

**Published:** 2014-01-09

**Authors:** Shota Oikawa, Hiroko Nomura, Miki Nishio, Rina Nagata, Tadayoshi Hata

**Affiliations:** ^1^Division of Clinical Pathophysiology, Graduate School of Health Sciences, Fujita Health University, 1-98 Dengakugakubo Kutsukake, Toyoake, Japan; ^2^Division of Pharmacology, Graduate School of Health Sciences, Fujita Health University, Japan

## Abstract

*Objectives*. Doxapram hydrochloride is a respiratory stimulant that has an inhibitory effect on myocardial IK1 potassium channels and is thought to increase membrane instability and excitability in myocardial cells. We examined the arrhythmogenic effects of doxapram hydrochloride in a rat model of halothane adrenaline-induced arrhythmia. *Methods*. Thirteen female Wistar rats (12–14 weeks old) were used in the study. Animals were anesthetized with inhalation of halothane to permit observation of the effects of doxapram hydrochloride on halothane adrenaline-induced arrhythmia. Time-dependent changes in ECG repolarization characteristics (QT, QTc, JTp, JT, and Tp-e intervals) were studied. *Results*. Doxapram hydrochloride itself did not induce arrhythmia but did induce bidirectional ventricular tachycardia after addition of adrenaline. *Conclusion*. Drug-induced impairment of intracellular Ca^2+^ regulation caused BVT in the absence of genetic abnormalities in proteins in the sarcoplasmic reticulum.

## 1. Introduction

Doxapram hydrochloride is a respiratory stimulant that is mainly used for anesthesia awareness in adult patients and treatment of apneic episodes in low birth weight infants. The mechanism of action of doxapram hydrochloride involves stimulation of the respiratory center, that is, effects on K^+^ channels such as TASK-1 and -3 in carotid bodies, which depolarizes membrane potentials and increases secretion of neurotransmitters [1, 2]. However, the pharmacological action of doxapram hydrochloride in other organs has not been studied extensively.

Halothane increases myocardial sensitivity to adrenaline, and adrenaline administered under halothane anesthesia readily causes premature ventricular contraction, resulting in fatal ventricular fibrillation; therefore, this combination is used as an anesthetic-induced arrhythmic model [3, 4]. We have found that clinical doses of doxapram hydrochloride have no effect on the heart conduction system in rats [5], while doxapram hydrochloride at high doses prolongs the myocardial repolarization interval and causes deterioration of halothane adrenaline-induced arrhythmia. In this study, we focused on doxapram hydrochloride-induced bidirectional ventricular tachycardia (BVT) that is a rare entity occurring in conditions associated with intracellular calcium overload. Delayed afterdepolarizations occurring in different zones of the conduction system are thought to best explain its mechanism [6]. We studied changes in ECG repolarization characteristics before and after onset of tachycardia.

## 2. Methods

### 2.1. Animals

Female Wistar Sprague-Dawley (SD) rats (age: 12–14 weeks, mean body weight: 220 ± 58 g, and *n* = 13) were used in the study. Animals were anesthetized with inhalation of halothane (1.2%) using an anesthetizer for small animals (Bio Research Center, Nagoya, Japan) and experiments were conducted in spontaneously breathing animals fixed in a supine position. Rectal temperature was monitored while using a BWT-100A body temperature maintenance system (Bio Research Center, Nagoya, Japan) to keep the body temperature at 35°C. Drugs were administered using a catheter placed in the femoral vein. CM5-lead ECG was continuously recorded throughout the experiment at a 1000 Hz sampling rate using a MP150 biosignal recorder (BIOPAC Systems, Goleta, CA, USA) with subcutaneous-attached disposable electrodes. The animal experiments were approved by the Institutional Review Board of Fujita Health University and conducted in compliance with Animal Welfare Regulations. Animal feeding and carcass handling were conducted with appropriate ethical considerations.

### 2.2. ECG Analysis

**Figure 1:**
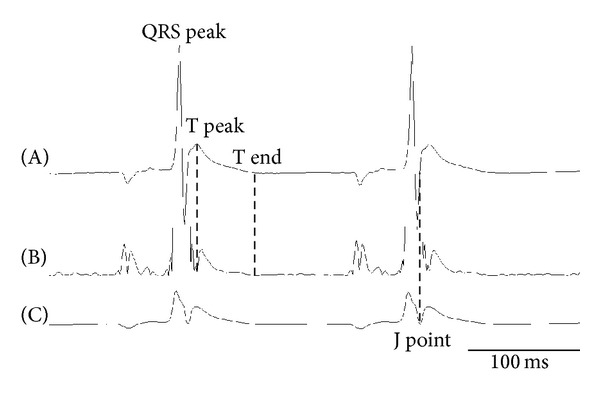
ECG interval analysis. Upper trace (A) is an original ECG and (B) is a form of the absolute value processing and after the first differential. (C) is a form after the smoothing procedure. Representative trace of lead CM5. T peak: peak of T wave, T end: end of T wave, and J point: onset of J wave.

**Figure 2:**
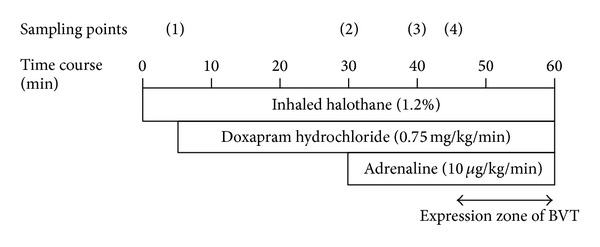
Schema of experimental protocol to study the effects of doxapram hydrochloride on adrenaline-induced arrhythmias in halothane anesthetized rat. The circled numbers (sampling points) denote the time of measuring ECG parameters shown in Table 1. BVT: bidirectional ventricular tachycardia.

ECG recordings were analyzed using AcqKnowledge 3.9.1 (BIOPAC Systems). P wave, QRS onset, QRS peak, QRS complex (J point), T peak, and T end were determined by linear derivation and the absolute values to measure the RR and QT intervals of the same heartbeats. Smoothing methods were used in order to determine the J point. Further, RR, PQ, QT, corrected QT (QTc, corrected by Fridericia equation), corrected JT (JTc), JT peak (JTp), and T peak-T end (Tp-e) were measured (Figure 1). Based on these values, JTp/JT and Tp-e/QT were estimated as characteristics of myocardial repolarization. As shown in the protocol (Figure 2), parameters for 10 continuous beats (narrow QRS complex) from each extracted point were estimated and the mean was used for analysis.

### 2.3. Exclusion Criteria

Experiments were terminated or data were excluded from the final analysis if bradycardia occurred prior to drug or vehicle administration. Initially, 17 rats were used in the study, but data from 4 animals were excluded due to cardiac or respiratory arrest before adrenaline administration.

### 2.4. Experimental Protocol

The study protocol is shown in Figure 2. Saline or drug solution was continuously administered using a catheter inserted in the right groin (the injection volume was adjusted to 0.7 mL with saline). Doxapram hydrochloride (0.75 mg/kg/min) was continuously administered from a side tube. Subsequently, adrenaline was administered at a dose of 10 *μ*g/kg/min. ECG recordings before administration of drugs at sampling point (1), 20 min after administration of doxapram hydrochloride at sampling point (2), at steady-state without arrhythmia after administration of adrenaline at sampling point (3), and immediately before the onset of BVT at sampling point (4) were used for analysis.

### 2.5. Drugs

Halothane (Takeda Pharmaceutical Company Limited, Japan), doxapram hydrochloride (Kissei Pharmaceutical Company Limited, Matsumoto, Japan), and adrenaline (Daiichi Sankyo Company Limited, Japan) were used in the study. Doxapram hydrochloride and adrenaline were diluted in saline (Otsuka Pharmaceutical Company Limited, Japan).

### 2.6. Statistics

ECG parameters are shown as the mean ± standard deviation. Statistical analysis was conducted using JMP Statistical Analysis Software (SAS Institute Inc., Cary, NC, USA). After testing by ANOVA, a Tukey-Kramer multiple comparisons test (HSD test) was conducted with a significance level of 0.05.

## 3. Results

### 3.1. Changes in ECG Parameters

As shown in Table 1, doxapram hydrochloride shortened the time course and RR interval and prolonged QTc and JTpc. The RR interval significantly decreased from 213.8 ± 13.5 ms to 188.1 ± 17.3 ms at 20 min after administration. In contrast, QTc and JTpc were prolonged from 141.8 ± 16.6 ms to 148.0 ± 13.0 ms and from 11.8 ± 4.8 ms to 17.5 ± 4.8 ms, respectively. However, single administration of doxapram hydrochloride did not induce arrhythmia (0 out of 13 rats). Sporadic premature ventricular contraction occurred several minutes after administration of adrenaline (13 out of 13 rats). Significant prolongation of the JTp time in the ascending limb of the T wave occurred 10 minutes later at sampling point (3). Continuous premature ventricular contraction (ventricular tachycardia) was also observed and this gradually complicated with BVT. In analysis of the narrow QRS complex wave immediately before continuous ventricular tachycardia at sampling point (4), JTc and JTpc significantly increased. ECGs at baseline (A), after administration of doxapram hydrochloride (B) and adrenaline (C), before onset of ventricular tachycardia (D), and with BVT and VT (E) after adrenaline administration are shown in Figure 2.

### 3.2. Changes in Characteristic Ratios

**Figure 3:**
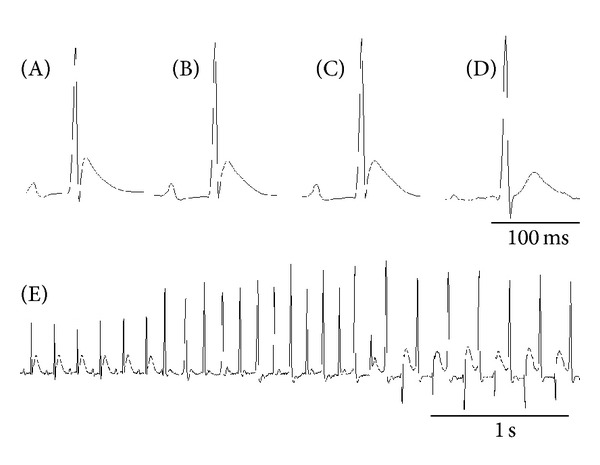
Characteristics of electrocardiographic changes with representative agents. (A) Control, (B) 10 min after doxapram hydrochloride administration, (C) 10 min after adrenaline administration, (D) just before the ventricular tachycardia, and (E) continuous electrocardiogram are migrating to bidirectional ventricular tachycardia and premature ventricular contraction from normal sinus rhythm.

**Figure 4:**
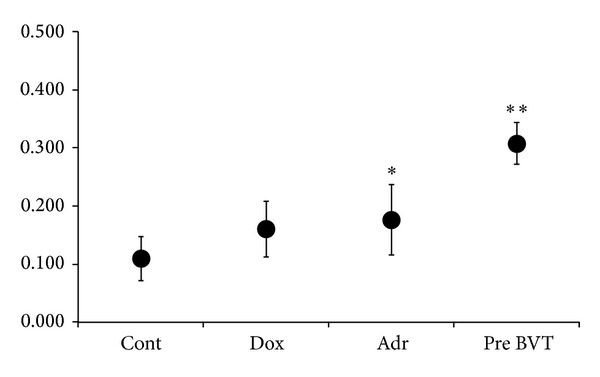
Effects of doxapram hydrochloride and adrenaline on the JTp/JT ratios. Results are presented as mean JTp/JT ± SD. *P < 0.05 (compared with control), **P < 0.05 (compared with all others) *n* = 13. Cont; control, Dox; doxapram hydrochloride, Adr; adrenaline, pre BVT; just before the ventricular tachycardia).

Changes in the slope and time from the J point, which is the ascending limb of the T wave, to the T wave peak were observed and JTp/JT, a characteristic ratio of repolarization, increased until immediately before onset of BVT. JTp/JT increased from 0.110 ± 0.038 at baseline to 0.160 ± 0.048, 20 min after administration of doxapram hydrochloride and significantly increased to 0.176 ± 0.060 10 min after administration of adrenaline (Figure 3). Thereafter, JTp/JT increased to 0.307 ± 0.036 immediately before onset of BVT (Figure 4).

## 4. Discussion

The results of the study show that doxapram hydrochloride causes deterioration of halothane adrenaline-induced arrhythmia and induces BVT in rats. Immediately before onset of BVT, there was an increase in JTp/JT, a characteristic of myocardial repolarization. Doxapram hydrochloride [1, 2] is a respiratory stimulant that inhibits K^+^ channels, including the IK1 channel in myocardial cells. TASK-1 and TASK-3 channels, which are sensitive to doxapram hydrochloride, are found in myocardial cells and provide the background current to maintain the resting membrane potential [7, 8]. Thus, doxapram hydrochloride destabilizes the resting membrane potential of the myocardial membrane. In our preliminary study, only sporadic premature ventricular contraction was induced by single administration of adrenaline after anesthesia with halothane. However, after pretreatment with doxapram hydrochloride, both BVT and onset of ventricular tachycardia occurred after premature ventricular contraction. This result shows that excitability due to instability of the resting membrane potential and irregular Ca^2+^ influx into cells increases arrhythmogenic effects.

Adrenaline-sensitive ventricular tachycardia has been linked to a genetic abnormality in a ryanodine receptor in the sarcoplasmic reticulum causing Ca^2+^ release and Ca^2+^ overload-induced delayed afterdepolarization (DAD) [9]. An arrhythmogenic substrate can also be induced by genetic mutation of calsequestrin, a protein involved in Ca^2+^ storage [10, 11]. In contrast, the pathogenic mechanism of BVT showing a specific waveform is not clearly understood. This arrhythmia may develop in patients with digitalis intoxication, that is, due to an intracellular Ca^2+^ increase by Na^+^-K^+^ ATPase inhibition [12, 13], and in patients with catecholamine-sensitive ventricular tachycardia who have a genetic mutation of the ryanodine receptor [14]. This indicates that intracellular Ca^2+^ overload associated with sarcoplasmic reticulum dysfunction can induce BVT. Administration of adrenaline in halothane anesthesia has been experimentally and clinically shown to induce premature ventricular contraction and ventricular tachycardia [15, 16]. The pathogenic mechanism is thought to be DAD associated with abnormal intracellular Ca^2+^ regulation, but the myocardial repolarization interval has not been examined in detail using body surface ECG.

We analyzed the characteristics of myocardial repolarization in this study, with a focus on the QT interval. QTc and JTc were prolonged by doxapram hydrochloride and were further prolonged, rather than shortened, by addition of adrenaline. JTp/JT, a characteristic of myocardial repolarization, showed a stepwise increase after administration of doxapram hydrochloride, after administration of adrenaline, and immediately before onset of BVT. An increasing number of basic and clinical studies have suggested that the interval from the peak to the end of the electrocardiographic T wave (Tp-e) and Tp-e/QT ratio may correspond to the transmural dispersion of repolarization and the increments of those parameters are associated with malignant reentrant ventricular arrhythmias [17]. In our study, there was no observation the increment of Tp-e and Tp-e/QT ratios just before the ventricular tachycardia. On the other hand, an increase in sympathicotonia in mammals is considered to shorten the ascending limb of the T wave with enhanced activation and inactivation of Ca^2+^ currents and to shorten the QT interval with increased outward current IKr [18, 19]. In this study, however, halothane decreased the repolarization reserve [20] and the JTp and QT intervals were prolonged, despite stimulation of the cardiac sympathetic nerve by adrenaline. This outcome is consistent with delayed inactivation of Ca^2+^ influx by an increased intracellular Ca^2+^-induced long QT [21], as shown in a rat arrhythmia model by Lin et al. Therefore, we suggest that our observation of prolongation of the ascending limb of the T wave was due to dysregulation of Ca^2+^ current, a pathogenic status of intracellular Ca^2+^ overload, including decreased K^+^ efflux that regulates the action potential duration.

The results of this study do not directly reveal the pathogenic mechanism of bidirectional ventricular arrhythmia, but it appears that the prolonged cardiac action potential including the conduction system is due to a prolonged JTp interval. Therefore, DAD and the triggered activity appear to occur alternately between the anterior and posterior fascicles [13, 22]. Doxapram hydrochloride administration increased the heart rate in rats, indicating that T wave alternans, an indicator for temporal heterogeneity, should be used for evaluation. A more complete understanding of this phenomenon will require evaluation of ionic currents in *in vitro* studies and direct examination of modulation of intracellular calcium kinetics. Clinically, it is rarely encountered in BVT. Doxapram hydrochloride poisoning, however, causes the intracellular calcium dysregulation to induce arrhythmia.

## 5. Conclusion

Doxapram hydrochloride administration led to deterioration of halothane adrenaline-induced arrhythmia and induced ventricular tachycardia, including bidirectional ventricular tachycardia. Thus, experimentally induced Ca^2+^ overload in myocardial cells can induce specific bidirectional ventricular tachycardia in the absence of a gene mutation in proteins in the sarcoplasmic reticulum.

## Figures and Tables

**Table 1:** Changes in ECG parameters.

	Cont	Dox	Adr	Pre BVT
RR	213.8 ± 13.5	188.8 ± 10.0*	188.1 ± 17.3*	166.0 ± 10.7**
QT	85.0 ± 10.2	84.9 ± 7.8	89.1 ± 7.5	94.4 ± 10.6
QTc	141.8 ± 16.6	148.0 ± 13.0	155.5 ± 11.6	172.1 ± 20.1*
JT	64.0 ± 11.3	63.5 ± 8.5	67.4 ± 9.5	72.0 ± 10.6
JTc	106.4 ± 18.7	110.6 ± 14.4	117.6 ± 14.9	131.3 ± 19.7*
JTp	7.1 ± 2.9	10.1 ± 2.8	11.8 ± 3.9*	22.2 ± 4.6**
JTpc	11.8 ± 4.8	17.5 ± 4.8	20.6 ± 7.1*	40.5 ± 8.4**
Tp-e	56.9 ± 10.1	53.4 ± 7.9	55.6 ± 9.4	49.8 ± 7.1
Tp-ec	95.0 ± 16.1	93.1 ± 13.5	97.0 ± 14.7	90.8 ± 13.4

Values are shown as the mean (ms) ± SD. *P < 0.05 (compared with control); **P < 0.05 (compared with all others) *n* = 13.

Cont: control, Dox: doxapram hydrochloride, Adr: adrenaline, and pre BVT: just before the ventricular tachycardia (calculated from the narrow QRS complex). Tp-e: T peak to T end interval, Tp-ec: corrected Tp-e interval.
